# Mechanisms during Strain Rate-Dependent Crack Propagation of Copper Nanowires Containing Edge Cracks

**DOI:** 10.3390/nano13071231

**Published:** 2023-03-30

**Authors:** Jinjie Zhou, Fankai Xian, Jinchuan Shen

**Affiliations:** School of Mechanical Engineering, North University of China, Taiyuan 030051, China

**Keywords:** molecular dynamics, crack propagation, plastic deformation, crack depth, strain rate effect

## Abstract

The crack propagation mechanism of Cu nanowires is investigated by using molecular dynamics methods. The microstructural evolution of crack propagation at different strain rates and crack depths is analyzed. Meanwhile, the stress intensity factor at the crack tip during crack propagation is calculated to describe the crack propagation process of Cu nanowires under each condition. The simulation results show that the competition between lattice recovery and dislocation multiplication determines the crack propagation mode. Lattice recovery dominates the plastic deformation of Cu nanowires at low strain rates, and the crack propagation mode is shear fracture. With the increase in strain rate, the plastic deformation mechanism gradually changes from lattice recovery to dislocation multiplication, which makes the crack propagation change from shear fracture to ductile fracture. Interestingly, the crack propagation mechanism varies with crack depth. The deeper the preset crack of Cu nanowires, the weaker the deformation resistance, and the more likely the crack propagation is accompanied.

## 1. Introduction

Metal nanowires are expected to play an important role in future nanoscale mechanical, optical, and electrical devices due to their unique structure, properties, and the great potential they show in a variety of applications such as molecular electronics and nanoelectromechanical systems (NEMS) [1,2]. In recent years, various nanodevices have been developed from nanowires, such as nano-laser [3,4] and quantized conductance atomic switches [5]. Since the ratio of surface area to volume of nanowires is greatly increased at the nanoscale, this enhances the surface effect and makes the mechanical properties of metal nanowires differ significantly from those of bulk metals [6,7]. This has led to extensive research on small-scale high-strength material systems and phenomena in recent years.

MD simulation, as an atomic simulation technique, plays an important role in describing the plastic deformation and crack propagation processes of metallic materials under mechanical loading and becomes an effective method for evaluating the deformation and fracture behavior of nanomaterials [8]. Many researchers have applied molecular dynamics (MD) simulations of metal nanowire structures, plastic deformation and fracture models, strain rate effects, size effects, crystal orientation effects on the yield stress and its deformation mechanism.Strain rate is known to be a crucial loading parameter determining metal response, from creep at very low strain rates to fragmentation or spallation in the regime of shock-wave loading. Phase transformation [9] and amorphization [10,11] occur at high strain rates (above $10^{10}{\;s}^{-1}$). At relatively low strain rates ($10^{6}–10^{9}{\;s}^{-1}$), when the strain rate exceeds $10^{8}{\;s}^{-1}$, the yield stress of metal nanowires increases rapidly with increasing strain rate and differs significantly from that of bulk metals. Plastic deformation at a strain rate of $10^{8}{\;s}^{-1}$ is dominated by dislocation slip and twinning. Lin et al. [12] investigated the tensile behavior of Cu nanowires at $10^{7}\;–10^{9}{\;s}^{-1}$ strain rates and showed that at lower strain rates ($7\times 10^{7}{\;s}^{-1}$), the number of dislocation nucleation is relatively low, which easily leads to single slip of dislocations and thus completes the yielding behavior, resulting in low yield stresses. The higher the strain rate, the more pronounced the high yield stress is, making it easier to nucleate dislocations and more likely to activate multiple slips of dislocations at the same time. Hansson et al. [13] investigated the combined effect of strain rate on the initial defect-free square cross-section nanocrystalline copper bundles under displacement-controlled tensile loading. The selected strain rate range was $0.5\times 10^{8}{\;s}^{-1}–5\times 10^{8}{\;s}^{-1}$. The strain rate has a significant effect on the plastic development of the beam. Increasing the strain rate reduces the onset strain and localizes the plasticity near the ends, which leads to rupture. The mechanism of the effect of strain rate on the tensile behavior of Cu nanowires was investigated based on the simulations of Zhao et al. [14]. Slip mode, mixed mode, and necking mode were observed at lower, moderate, and higher rates, respectively. The fracture mechanism is due to competition between lattice recovery and dislocation multiplication. Dislocation multiplication gradually dominates the deformation as the strain rate increases. Rajaprakash et al. [15] reported in situ scanning electron microscopy (SEM) tensile tests of bicrystalline silver nanowires, where a significant brittle-ductile failure mode transition was observed at a threshold strain rate of 0.2/s. Previous results show that phase transformation and amorphization occur at high strain rates (above $10^{10}{\;s}^{-1}$), and at lower strain rates ($10^{6}\;–10^{9}{\;s}^{-1}$), dominant plastic deformation occurs with dislocations and twins, and dislocations are more likely to nucleate as the strain rate increases.

On the other hand, microscopic defect structures such as microcracks are easily generated during the preparation and use of nanowires, which have a great impact on the mechanical properties of the materials. In order to improve the safety and reliability of equipment operation, it is necessary to analyze the microstructural damage of single-crystal nanomaterials and understand the failure mechanism of nanomaterials. Many studies have been conducted on cracks in FCC materials at the atomic scale. Singh et al. [16] investigated the effect of fracture behavior of pre-cracked FCC single crystals under tensile loading, for the plastic deformation mechanism of dislocation emission and interaction of FCC Cu, Ni and Al single crystals at the crack front. For materials with high stable stacking fault energy, sessile dislocations formed at the extended crack front (such as Stair-rod dislocations, Hirth locks, and Frank PDs dislocations) lead to a decrease in fracture toughness by increasing the local shear stress along the slip plane. However, for materials with low stable stacking fault energy, it is mainly the evolution of sliding dislocations (Shockley dislocations) and twinning at the crack front that lead to an increase in fracture toughness. Rajput et al. [17] investigated the crack extension in copper single crystals, and two different crystal orientations (001) [$\bar{1}$10] and (001) [010] were chosen. For the copper single crystal with the crack tip orientation of (001) [010], the stress is concentrated at the crack tip, and the crack extension by decoupling occurs due to the lack of dislocation activity at the crack tip. For copper single crystals with a crack tip orientation of (001) [$\bar{1}$10], continuous emission of dislocations at the crack tip results in reduced crack tip passivation and stress concentration. The effect of different crack depth on the crack extension behavior of FCC materials was investigated by Qiu et al. [18]. For tensile processes with different crack lengths, the simulation results of the complete substrate are similar to those with small crack lengths. Moreover, the complete substrate has the largest Young’s modulus. As the crack length increases, the Young’s modulus decreases. Velilla-Díaz et al. [19] performed molecular dynamics simulations on aluminum nanocrystals. Atomic models of single and bicrystals were developed considering eleven different crack lengths. The results show that the effect of grain boundaries on fracture toughness can increase the fracture toughness of bicrystals by nearly four times.

However, the effect of strain rate on the crack propagation of nanowires is rarely studied. In this study, we apply MD to simulate the deformation mechanism and crack expansion mode of Cu nanowires with initial cracks under Type I loading mode. The relationship between mechanical response and the crack propagation mode of Cu nanowires is established. The effect of crack depth and strain rate on the crack propagation mechanism is discussed. The crack propagation of nanowires is explained by the stress intensity factor. This study provides theoretical guidance for understanding the crack propagation and fracture behavior of Cu nanowires.

## 2. Models and Methods

Interatomic potential is very important for the accuracy of simulation results in molecular dynamics simulation. In this model, all simulations were performed using the embedded atomic method (EAM) for interatomic potential developed by Mishin et al. [20]. The EAM potential takes into account the local electron density and can effectively describe the bonding in metallic systems. It can accurately predict lattice properties, point and extended defects, various structural energies, and transition paths. The total energy of the system is given by Equation (1).

(1) $$E_{r}=\sum_{i=1}^{N}F_{i}\left(\rho_{i}\right)+\frac{1}{2}\sum_{\mathrm{ij},i\neq j}\Phi_{\mathrm{ij}}\left(r_{\mathrm{ij}}\right)$$

(2) $$\rho_{i}=\sum_{j\neq i}f_{j}\left(r_{\mathrm{ij}}\right)$$

The total energy of the system consists of two parts: the embedded energy, which is $F_{i}\left(\rho_{i}\right)$ in Equation (1), and the pair potential, which represents the interaction between atoms. The sum of the densities of the electron clouds created by the electrons outside the nuclei of the other atoms at the ith atom is $\rho_{i}$. The distance between the ith atom and the jth atom is $r_{\mathrm{ij}}$, and the fitted distribution function for the electron density is $f_{j}\left(r_{\mathrm{ij}}\right)$.

The definition of stress in atomic simulations is different from the concept of continuous stress. In this study, the well-known and commonly used definition of Virial stress is used [21]. Virial stress at the atomic scale is equivalent to continuous Cauchy stress [22]. This stress consists of two components, the potential energy component, and the kinetic energy component, defined as:

(3) $$\sigma_{\alpha \beta }=\frac{1}{v}\sum_{i}[\frac{1}{2}\sum_{j=1}^{N}(r_{\alpha }^{j}-r_{\alpha }^{i})f_{\beta }^{\mathrm{ij}}-m^{i}v_{\alpha }^{i}v_{\beta }^{i}]$$

where the subscripts α and β denote the Cartesian components, and V denotes the total volume of the system. Here, atom i has N neighbors of atom j. The α components at the positions of atom i and atom j are $r_{\alpha }^{i}$ and $r_{\alpha }^{j}$ respectively. The β-directional force of atom j on atom i is $f_{\beta }^{\mathrm{ij}}$. The mass of atom i is $m^{i}$. The velocities of atom i along the α and β directions are $v_{\alpha }^{i}$ and $v_{\beta }^{i}$ respectively. To roughly calculate the local stress field of the system, the “atomic stress” of each atom in the model is used to present a snapshot of the MD calculated results. Here, “atomic stress” corresponds to the term in square brackets in Equation (3) and has the unit of stress × volume.

Molecular dynamics simulations use the Large Atomic/Molecular Massively Parallel Simulator (LAMMPS) [23]. To characterize microscopic phenomena such as dislocations, twinning, and phase transitions in the material during stretching, the dislocations are extracted using the Dislocation Extraction Algorithm (DXA) [24], which is implemented in OVITO [25]. Using the centrosymmetric parameter method (CSP) [26], the centrosymmetric parameter of atom j is:

(4) $$\rho_{j}=\sum_{j=1}^{6}{\left|{\underset{R}{\to }}_{j}+{\underset{R}{\to }}_{j+6}\right|}^{2}$$

where ${\underset{R}{\to }}_{j}$ and ${\underset{R}{\to }}_{j+6}$ are the six-pair vectors of the relatively nearest atoms in FCC single-crystal Cu. The crystal structure defect is defined as: when CSP > 1, the dislocation type is shown as all dislocations in the model. When 1 < CSP < 4, dislocation nucleation and defect expansion by thermal fluctuations and external stresses in dislocation nucleation are shown. When 4 < CSP < 11, it is shown as stacking layer dislocations. When 11 < CSP < 19, it is shown as a complete surface. When CSP > 19, it is shown as dislocation steps. A larger value of the centrosymmetry parameter indicates a higher degree of a mismatch for that atom.

**Figure 1:**
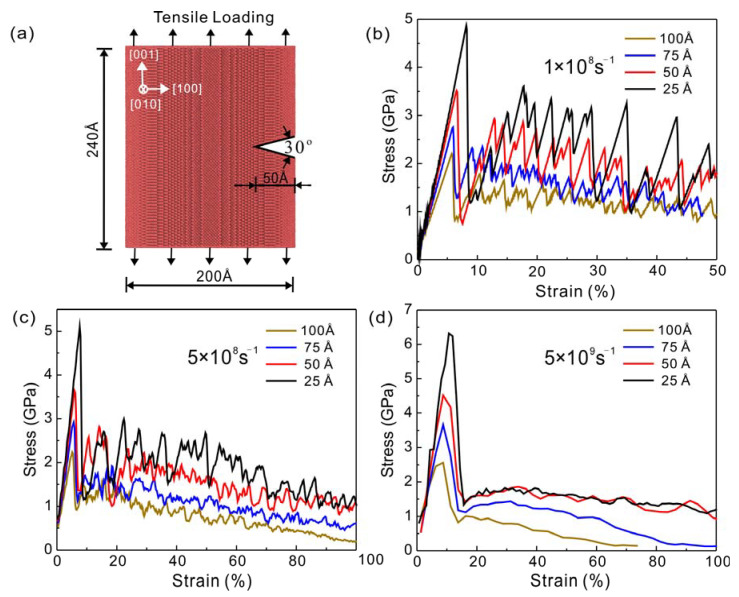
(**a**) Schematic diagram of a Cu nanowire with a 50 Å edge crack. The crack plane is located in the (001) plane, and the leading edge of the crack is along the [010] direction. Stress-strain curves of Cu nanowires with (001) [010] four different depths of edge cracks under type I loading conditions: (**b**) $\;1\times 10^{8}{\;s}^{-1}$, (**c**) $\;5\times 10^{8}{\;s}^{-1}$, (**d**) $\;5\times 10^{9\;}s^{-1}$.

We determine the fracture toughness based on the energy theory of fracture mechanics and using the thermodynamic integral (over the stress-strain curve during crack extension). During tensile loading, the internal stresses of a system increase from zero, together with the associated mechanical energy P. When the crack starts to propagate, the stored mechanical energy is released to create two new surfaces. Based on the energy theory of fracture mechanics, the Helmholtz free energy F can take the form of the mechanical energy P under the NVT ensemble. The critical strain energy release rate is defined as the energy released per unit area of crack (XY plane in Figure 1) during crack propagation, which is an important measure of fracture toughness based on the Griffith’s theory [27,28]:

(5) $$G_{C}=\left(\frac{L_{x}L_{y}}{\Delta A_{\infty }}\right)\int_{L_{z}^{0}}^{L_{z}^{\max}}\sigma_{\mathrm{zz}}^{*}{\mathrm{dL}}_{z}$$

where $\;\Delta A_{\infty }$ is the total area of cracks produced after a complete fracture. The effective stress along the z-direction is $\sigma_{\mathrm{zz}}^{*}$, and the dimensions of the simulation frame in the x, y, and z directions are $L_{x}$, $L_{y}$, and $L_{z}$ respectively.

An important measure of fracture toughness is the critical Stress Intensity Factor $K_{\mathrm{IC}}$, which is commonly used in engineering applications for type I fractures. $K_{\mathrm{IC}}$ represents the ratio between the critical stress at the crack tip before crack propagation and the external loading stress away from the crack tip. $K_{\mathrm{IC}}$ can be estimated from $G_{c}$ based on Irwin’s formula for the plane strain loading conditions considered here and the isotropic assumption [29]:

(6) $$K_{\mathrm{IC}}={\left(\frac{G_{c}E}{1-\nu^{2}}\right)}^{1/2}\;$$

where E is Young’s modulus of the model and ν is Poisson’s ratio of Cu at 300 K, here ν taken as 0.324 [30].

The radius of the cylindrical Cu model is 100 Å and the dimension in the z-direction is 240 Å. The edge crack depths of the Cu nanowires were set to 25 Å, 50 Å, 75 Å and 100 Å, respectively. The Cu nanowires with different crack depths are simulated at three different strain rates of $1\times 10^{8}{\;s}^{-1}$, $5\times 10^{8}{\;s}^{-1}$, and $5\times 10^{9}{\;s}^{-1}$. The crack was set by deleting the triangular region with an angle of 30°, and the lattice parameter a of Cu is 3.61 Å. The initial configuration of the nanowires with a crack depth of 50 Å is shown in Figure 1a. The models are [100], [010], and [001] in the X, Y, and Z directions, respectively, by applying periodic boundary conditions in the [001] direction and free boundary conditions in the [100] and [010] directions.

The model was placed in a Nose/Hoover heat bath to relax at 300 K for 1 ps so that the energy and stress of the model were at a minimum level, minimizing the effect of internal stress on the Cu nanowires. The simulated system is NVT with a time step of 1 fs. It is then stretched at a uniform speed along the z-axis until it stretches enough to fracture.

## 3. Results and Discussion

### 3.1. Stress-Strain Behavior

The crack propagation behavior of Cu nanowires at different strain rates was simulated by the molecular dynamics method. As shown in Figure 1b–d, in the elastic phase, the curve is nearly linear, the larger the crack depth, the smaller the slope of the elastic phase, the smaller the elastic modulus value, and will yield under lower strain. The stress-strain curves of Cu nanowires at 1 × 10^8^ s^−1^ strain rate are shown in Figure 1b. The stress of Cu nanowires at each crack depth increases linearly with strain at the initial stage and then decreases abruptly after reaching the peak stress, at which point the peak stress value is the yield stress. The stress-strain curves at a strain rate of 5 × 10^8^ s^−1^ are shown in Figure 1c. At this strain rate, strain softening occurs after yielding. The stress values at 100 Å and 75 Å crack depths increase by about 0.8 GPa and then decrease uniformly. The flow stress of the 100 A crack depth model continues to decrease until 0 GPa, and the crack propagates along the direction of the prefabricated crack tip until fracture. The stress-strain curve of the 75 Å crack depth model slows down the rate of stress decrease after ε = 68.63%, and its model crack tip propagates first and then passivates. The stress-strain curves for the 50 Å and 25 Å crack depth models showed a higher increase in stress values of about 2 GPa during the hardening phase, and the rate of decrease in stress values slowed down after ε = 60%, and the cracks did not expand in both models. The stress-strain curves of Cu nanowires at a strain rate of 5 × 10^9^ s^−1^ are shown in Figure 1d. The flow stress at 100 Å crack depth model decreases rapidly with increasing strain and is close to fracturing at 60% strain, while the flow stress at 75 Å crack depth model shows a small increase and then decrease (ε = 16.28% to 31.28%) and is close to fracturing at 100% strain. The stress-strain curves for 50 Å and 25 Å crack depth model show similar trends with a smooth decrease in flow stress.

**Figure 2:**
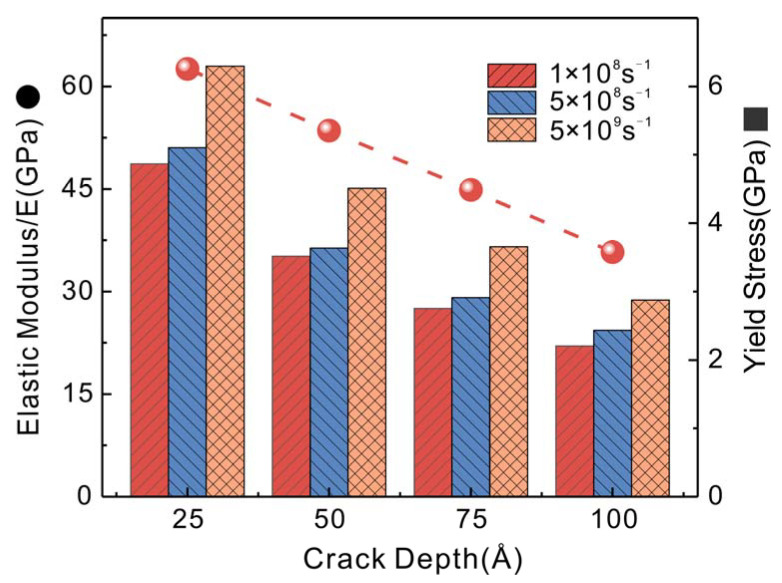
Variation of elastic modulus and yield stress with crack depth at different strain rates.

The elastic modulus and yield stress of Cu nanowires are important parameters to measure their mechanical properties. The variation of elastic modulus and yield stress with strain rate at different crack depths is shown in Figure 2. As the crack depth increases, the elastic modulus and yield stress decrease significantly. The significant decrease in elastic modulus indicates that the ability of Cu nanowires to resist deformation decreases with the increase in crack depth. The yield stress increased with increasing strain rate, while the elastic modulus of Cu nanowires was almost constant at different strain rates.

### 3.2. Deformation and Fracture Modes of Cu Nanowires

**Figure 3:**
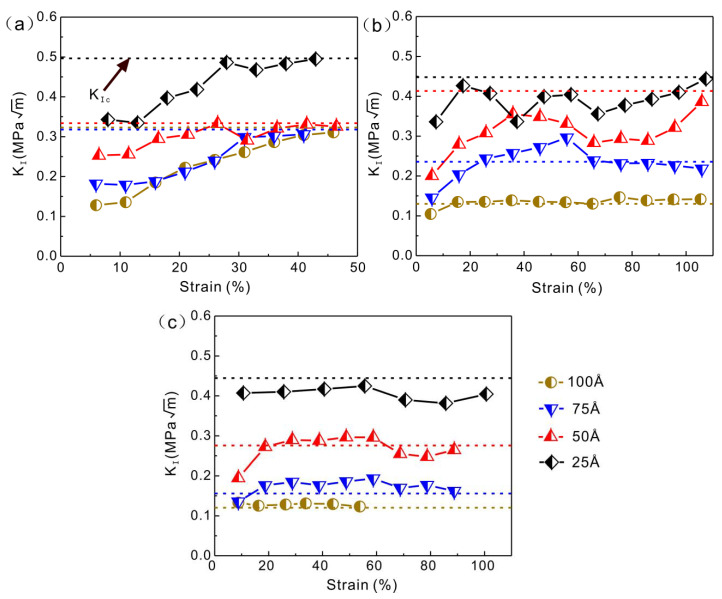
Stress intensity factor and critical stress intensity factor (dashed line) for Cu nanowires with different initial crack depths (**a**) $\;1\times 10^{8\;}s^{-1}$, (**b**) $\;5\times 10^{8}{\;s}^{-1}$, (**c**) $\;5\times 10^{9}{\;s}^{-1}$.

The stress intensity factor at the crack tip changes dynamically during crack propagation. The crack will propagate only when the stress intensity factor at the crack tip is greater than the critical stress intensity factor [31]. When the stress intensity factor is less than the critical stress intensity factor, the crack propagation is stalled. Whether cracking can proceed to propagation is determined by judging the magnitude of the stress intensity factor and the critical stress intensity factor. The crack tip stress intensity factor ($K_{I}$) is calculated using Equations (5) and (6), depending on the location of the crack tip. Several strain nodes are selected, and the stress intensity factor near the crack tip is calculated and compared with the critical stress intensity factor. The calculated results are shown in Figure 3. At $1\times 10^{8\;}s^{-1}$ strain rate, the $K_{I}$ values are all smaller than $K_{\mathrm{IC}}$, indicating that the Cu nanowires with four crack depths cannot propagate along the crack tip. At $5\times 10^{8}{\;s}^{-1}$ strain rate, the $K_{I}$ value at 100 Å crack depth is consistently greater than $K_{\mathrm{IC}}$ and the crack continues to expand. At 75 Å crack depth, the $K_{I}$ value is less than $K_{\mathrm{IC}}$ after ε = 68.63% and the crack is blunted after propagation. At 50 Å and 25 Å crack depth, the $K_{I}$ value is consistently less than $K_{\mathrm{IC}}$ and the crack fails to propagate. At $5\times 10^{9}{\;s}^{-1}$, the $K_{I}$ value is greater than $K_{\mathrm{IC}}$ at 100 Å and 75 Å crack depth. At 50 Å crack depth, after ε = 62.28%, the $K_{I}$ value is less than $K_{\mathrm{IC}}$. At 25 Å crack depth, the $K_{I}$ value is consistently less than $K_{\mathrm{IC}}$, and as the crack depth decreases, the crack propagation is gradually blocked.

**Figure 4:**
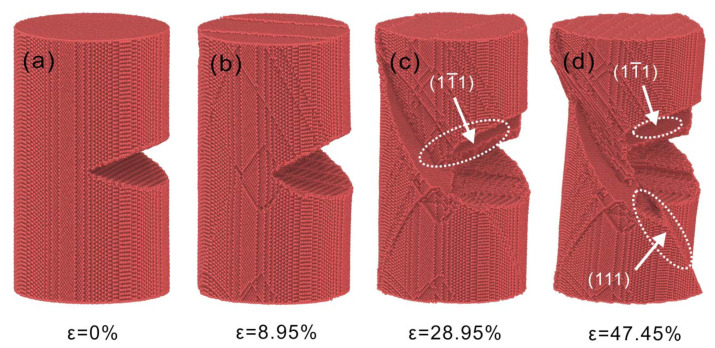
Microstructural evolution of Cu nanowires with 75 Å crack depth at $1\times 10^{8}{\;s}^{-1}$ strain rate. (**a**) ε = 0%, (**b**) ε = 8.95%, (**c**) ε = 28.95%, (**d**) ε = 47.45%.

The microscopic evolution of Cu nanowires with 75 Å crack depth at a strain rate of $1\times 10^{8\;}{\;s}^{-1}$ is shown in Figure 4. The Cu nanowires emit dislocations at the crack tip during yielding, causing a stress reduction, and after yielding. The stress begins to accumulate and cause shear deformation on the $\left(1\bar{1}1\right)$ dense row surface at ε = 10.95%, and the shear deformation on the slip surface continues to expand, as shown in Figure 4c. As the load proceeds, the single slip system is not enough to release the stress concentration, and the shear deformation begins on the plane (111), as shown in Figure 4d. Shear deformation along the dense row surface consumes a lot of energy, and the stress concentration at the crack tip is transferred to the slip surface. As a result, the shear deformation deviates from the direction of the preformed crack and exhibits a stepped expansion surface in the direction perpendicular to the crack tip plane. This process is dominated by the crystal lattice restoration; when the slip system activates and causes stress relief, the lattice deformation at the shear plane is reversed after the dislocation slips. The lattice reverts to the initial structure and properties of the crystal. The tensile process during crack propagation at $1\times 10^{8}{\;s}^{-1}$ strain rate is completely dominated by lattice recovery and exhibits shear deformation on the dense row surface.

**Figure 5:**
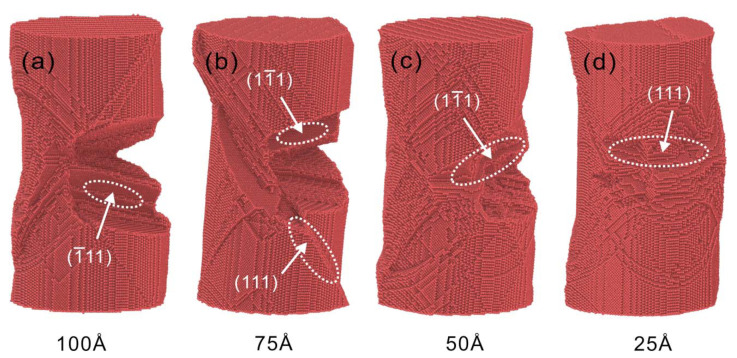
Atomic configurations for four depths of cracks at strain ε = 50% for a strain rate of $1\times 10^{8}{\;s}^{-1}$. (**a**) 100 Å, (**b**) 75 Å, (**c**) 50 Å, (**d**) 25 Å. The arrow in the figure shows the plane where the nanowire undergoes shear deformation.

The atomic configurations of Cu nanowires with four crack depths at $1\times 10^{8}{\;s}^{-1}$ strain rate are shown in Figure 5. The nanowires of all four crack depths could not be propagated along the crack tip at $1\times 10^{8}{\;s}^{-1}$ strain rate. The stress intensity factors of all four crack depths at this strain rate are less than the critical stress intensity factors, as shown in Figure 3a. The calculated results are consistent with the microscopic evolution results. The stress concentration is obvious at the crack tip with the increase of preset crack depth, which leads to a greater degree of shear deformation of Cu nanowires. The stress is in a state of accumulation and then release due to the breakage and bonding of interatomic bonds on the dense row surface, so the stress-strain curve shows sawtooth characteristics. As the crack depth decreases, the fracture between atomic bonds becomes more difficult, and plastic deformation requires greater stress, corresponding to greater stress fluctuations on the stress-strain curve.

**Figure 6:**
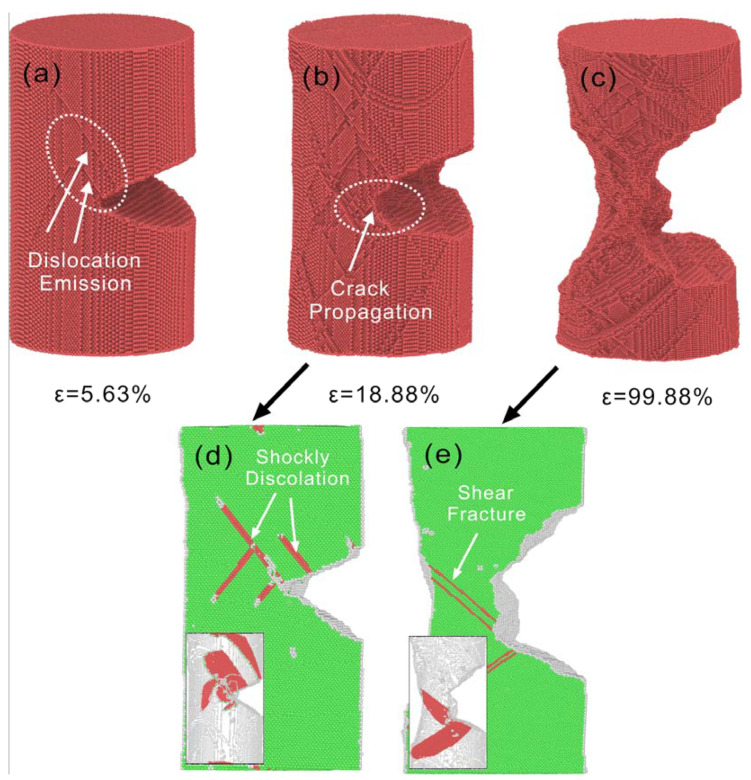
Atomic configuration of 75 Å crack depth Cu nanowires at strain rate $5\times 10^{8}{\;s}^{-1}$. (**a**) ε = 5.63%, (**b**) ε = 18.88%, (**c**) ε = 99.88%, (**d**,**e**) are the atomic configurations of (**b**,**c**) after CSP and DXA analysis, respectively.

The crack propagation process of Cu nanowires with 75 Å crack depth at $5\times 10^{8}{\;s}^{-1}$ strain rate is shown in Figure 6. The crack propagation occurs first at the edge of the predetermined crack, and the atomic bonds at the crack tip are broken in turn. At the same time, dislocation nucleation and dislocation slip at the crack tip allow the stresses in the Cu nanowires to be released, as shown in Figure 6d. As the loading continues, the crack tip dislocation nucleation and slip will reduce the crack tip stress level. At ε = 68.63%, the stacking fault at the crack tip hinders the crack propagation and blunts the crack tip, which is shown as the crack tip stress intensity factor is lower than the critical stress intensity factor, and the lack of stress concentration prevents dislocation emission from the crack tip. Subsequently, plastic deformation is dominated by lattice restoration. As shown in Figure 6e, the crack propagates along the surface of the stacking fault in the form of shear deformation.

The strain rate has an effect on the deformation mechanism during stretching, as shown by the competition between lattice recovery and dislocation multiplication. At low strain rate, the lattice recovery mechanism inhibits dislocation and plastic deformation occurs in shear mode on the sliding plane. At higher strain rates, the lattice recovery mechanism no longer dominates the plastic deformation of the crystal, and the plastic deformation is carried out in the way of dislocation slip.

**Figure 7:**
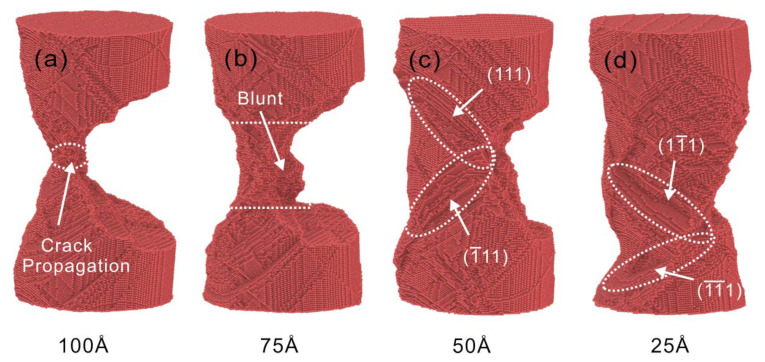
Atomic configuration of four deep cracks at $5\times 10^{8}{\;s}^{-1}$ strain rate. (**a**) 100 Å, (**b**) 75 Å, (**c**) 50 Å, (**d**) 25 Å.

The atomic structure of the crack propagation for a strain rate of $5\times 10^{8}{\;s}^{-1}$ at four depths is shown in Figure 7. The Cu nanowires with crack depths of 100 Å and 75 Å can propagate their cracks under tensile loading. Among them, 100 Å cracks propagate with the emission of dislocation until fracture and 75 Å cracks show passivation after propagation. Cracks at 50 Å and 25 Å crack depths cannot be for a strain rate of $5\times 10^{8}{\;s}^{-1}$ along the crack tip, and their plastic deformation mode is entirely shear deformation. The results of the stress intensity factor calculations are consistent with the microstructure evolution. Interestingly, crack depth is also related to the competition between the two mechanisms. The key is the degree of crystal stress concentration caused by external conditions. At a strain rate of $5\times 10^{8}{\;s}^{-1}$, the stress concentration is more severe at the crack tip of Cu nanowires at large crack depths, making the resistance to deformation weaker, and plastic deformation proceeds as dislocation slippage. The Cu nanowires at small crack depths resist deformation more strongly, and plastic deformation proceeds by lattice recovery.

**Figure 8:**
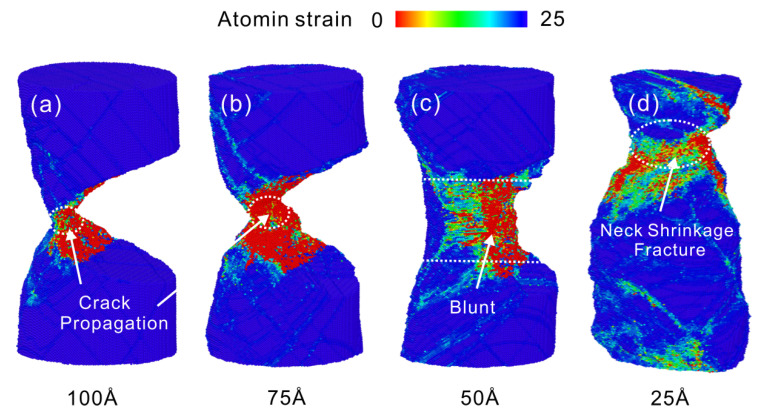
Atomic configuration for four depths of cracks at strain rate $5\times 10^{9}{\;s}^{-1}$. (**a**) 100 Å, (**b**) 75 Å, (**c**) 50 Å, (**d**) 25 Å.

The final atomic configurations of four crack depths under the strain rate of $5\times 10^{9}{\;s}^{-1}$, are shown in Figure 8. The cracks at 100 Å and 75 Å depths can expand rapidly until fracture. The crack at 50 Å propagates and then passivates. The crack at 25 Å depth fails to propagate and the fracture location changes. The propagation is consistent with the calculated trends of $K_{I}$ and $K_{\mathrm{IC}}$ in Figure 3c.

Cu nanowires with 100 Å and 75 Å crack depths emit dislocations at the crack tip along two slip planes $\left(\bar{1}11\right)$, $\left(111\right)$ and $\left(11\bar{1}\right)$, $\left(1\bar{1}1\right)$, respectively. The crack tip propagation process is continuously accompanied by the generation and slip of dislocations. 

As the crack depth decreases, more slip systems are initiated after yielding. The multiple slip systems interact to form dislocation junctions, thus providing strong resistance to dislocation motion. At the same time, the mobile dislocations are gradually transformed into pinned dislocations, and the total dislocation length gradually decreases. On the one hand, the dislocation plugging makes the stress increase. On the other hand, there are still dislocations that slip, making the stress decrease again. Dislocation plugging and dislocation slippage proceed simultaneously, and the stress values of Cu nanowires are kept in dynamic equilibrium. Under the action of dislocation plugging, the crack at a depth of 50 Å stops propagating. With the process of stretching, the cracks of Cu nanowires extend along the loading direction after passivation, as shown in Figure 8c. The crack passivation of 25 Å crack depth model is serious, and the stress concentration is transferred to the upper part of the model under the action of dislocation slip, as shown in Figure 8d.

**Figure 9:**
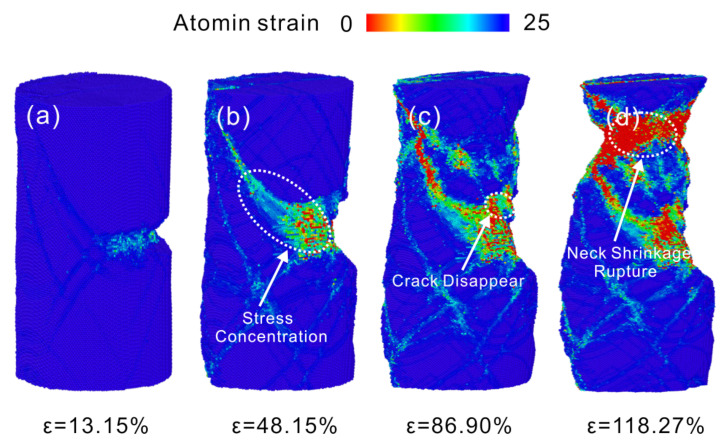
Atomic configuration of 25 Å crack depth Cu nanowires at $5\times 10^{9}{\;s}^{-1}$ strain rate. (**a**) ε = 13.15%, (**b**) ε = 48.15%, (**c**) ε = 86.90%, (**d**) ε = 118.27%.

The deformation process of Cu nanowires with 25 Å crack depth at a strain rate of $5\times 10^{9}{\;s}^{-1}$ is shown in Figure 9. After yielding, a large number of dislocations are emitted from the crack tip resulting in the slip and plugging of dislocations. As the load continues, the stress concentration at the crack tip shifts to face $\left(1\bar{1}1\right)$, as shown in Figure 9b. The subsequent plastic deformation process proceeds mainly along $\left(1\bar{1}1\right)$ side, causing the upper surface of the crack to disappear, as shown in Figure 9c. The stretching continues, and necking fracture occurs at the location shown in Figure 9d due to the formation of stress concentrations dominated by dislocation multiplication. Hanssonet al. [13] studied that increasing the strain rate reduces the initial strain and localizes the plasticity near the end, which leads to rupture, consistent with the results of necking fracture here.

The degree of passivation due to dislocation plugging after yielding varies for different crack sizes at $5\times 10^{9}{\;s}^{-1}$ strain rate. Smaller crack depths are less prone to stress concentration, and the initiation of multiple slip coefficients causes severe dislocation plugging and hinders nanowire crack propagation. The Cu nanowires with deeper cracks are prone to stress concentration at the crack tip, and only less slip system initiation is required to complete crack propagation. The deformation mode of Cu nanowires at each crack depth at this strain rate is entirely plastic deformation dominated by dislocation multiplication.

**Figure 10:**
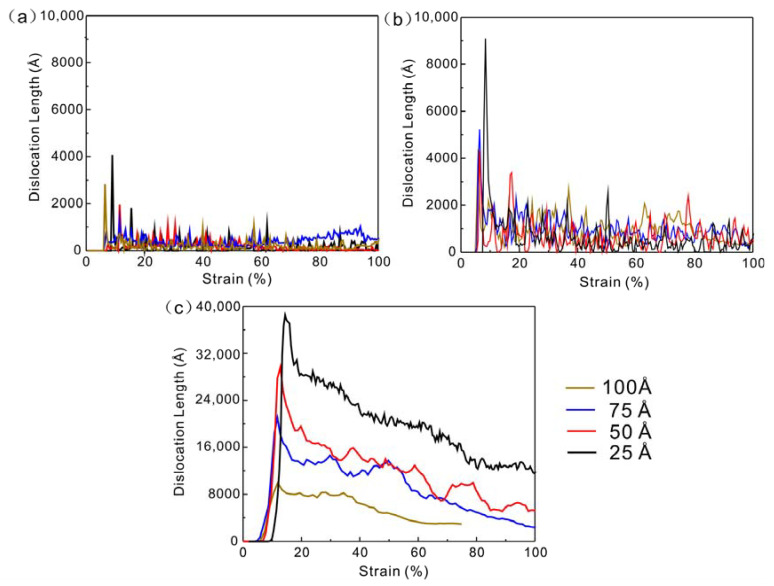
The variation of dislocation length with strain at different strain rates. (**a**) $\;1\times 10^{8}{\;s}^{-1}$, (**b**) $\;5\times 10^{8}{\;s}^{-1},\;$(**c**) $\;5\times 10^{9}{\;s}^{-1}$.

The variation of dislocation length with strain at different strain rates is shown in Figure 10. The length of dislocation line of Cu nanowires increases with the increase of strain rate. Lin et al. [12] showed that at lower strain rates ($7\times 10^{7}{\;s}^{-1}$), the number of dislocation nucleation is relatively low. The higher the strain rate, the more pronounced the high yield stress is, making it easier to nucleate dislocations and more likely to activate multiple slips of dislocations at the same time. This coincides with the model stress yielding results in Figure 1 and the dislocation density results in Figure 10. This indicates that the deformation mechanism of Cu nanowires gradually changes from lattice restoration to dislocation multiplication. Since lattice restoration involves the breaking and restoration of atomic bonds across the atomic plane, it consumes much more energy than dislocation slip. Therefore, the shear deformation dominated by lattice restoration is slow. On the contrary, crack propagation dominated by dislocation multiplication is more prone to fracture. The greater the depth of the Cu nanowire crack, the more likely it is to form a stress concentration and the more single the slip system initiated by crack expansion, prompting the crack to fracture in the set direction. When the slip system increases, the crossover and reaction between defects appear, which in turn passivates the crack tip.

## 4. Conclusions

In this paper, we investigate the different fracture behaviors of Cu nanowires. The (001) [010] edge cracking in Cu single crystal nanowires under type I loading conditions was considered using MD simulations. The deformation mechanism of Cu nanowires gradually changes from lattice recovery to dislocation multiplication with the change of strain rate. At $1\times 10^{8}{\;s}^{-1}$ strain rate, crack propagation at each depth is carried out in a lattice recovery-dominated shear fracture mode. At $5\times 10^{9}{\;s}^{-1}$ strain rate, the deformation process of each depth crack is dominated by dislocation multiplication. Interestingly, crack depth is also related to the competition between the two mechanisms. At $5\times 10^{8}{\;s}^{-1}$ tensile rate, with the increase of crack depth, the plastic deformation gradually changes from dislocation multiplication to lattice recovery dominated, which makes the crack propagation change from predetermined direction propagation to shear fracture along the slip plane direction, as the crack depth is closely related to the ability of the material to resist deformation. The greater the crack depth, the less the ability to resist deformation and the more single the slip system initiated when the crack expands. The key is the degree of crystal stress concentration caused by external conditions.

## Data Availability

The data presented in this study are available upon justified request.
